# High-Throughput Selection and Characterisation of Aptamers on Optical Next-Generation Sequencers

**DOI:** 10.3390/ijms22179202

**Published:** 2021-08-25

**Authors:** Alissa Drees, Markus Fischer

**Affiliations:** 1 Hamburg School of Food Science, Institute of Food Chemistry, University of Hamburg, Grindelallee 117, 20146 Hamburg, Germany; alissa.drees@chemie.uni-hamburg.de; 2 Center for Hybrid Nanostructures (CHyN), Department of Physics, University of Hamburg, Luruper Chaussee 149, 22761 Hamburg, Germany

**Keywords:** aptamers, next-generation sequencing, high-throughput assay, molecular biophysics, kinetics, HiTS–FLIP

## Abstract

Aptamers feature a number of advantages, compared to antibodies. However, their application has been limited so far, mainly because of the complex selection process. ‘High-throughput sequencing fluorescent ligand interaction profiling’ (HiTS–FLIP) significantly increases the selection efficiency and is consequently a very powerful and versatile technology for the selection of high-performance aptamers. It is the first experiment to allow the direct and quantitative measurement of the affinity and specificity of millions of aptamers simultaneously by harnessing the potential of optical next-generation sequencing platforms to perform fluorescence-based binding assays on the clusters displayed on the flow cells and determining their sequence and position in regular high-throughput sequencing. Many variants of the experiment have been developed that allow automation and in situ conversion of DNA clusters into base-modified DNA, RNA, peptides, and even proteins. In addition, the information from mutational assays, performed with HiTS–FLIP, provides deep insights into the relationship between the sequence, structure, and function of aptamers. This enables a detailed understanding of the sequence-specific rules that determine affinity, and thus, supports the evolution of aptamers. Current variants of the HiTS–FLIP experiment and its application in the field of aptamer selection, characterisation, and optimisation are presented in this review.

## 1. Introduction

Aptamers (latin *aptus*—suitable; greek *μέρος* (*méros*)—part) are synthetically produced, single-stranded nucleic acid oligomers (e.g., DNA or RNA) or peptides which fold into unique 3D structures and thereby can specifically bind target molecules [1,2]. The possible targets of aptamers comprise a wide diversity, ranging from ions [3] over small molecules [4], peptides and proteins [5,6], viruses [7,8], spores [9,10], bacterial cells [11], to eukaryotic cells [12].

While achieving similar affinity and specificity compared to antibodies, especially nucleic acid-based aptamers offer several advantages: (i) unlike antibodies produced in vivo, aptamers are synthesized entirely in vitro, an aspect desirable both for animal welfare reasons and for ensuring much greater batch stability; (ii) they are characterised by thermal stability, as well as (iii) the possibility to be adapted to a wide variety of environmental conditions (e.g., pH value, chemical composition, etc.); (iv) aptamers can be dried and easily reconstituted, which not only prolongs shelf life but also facilitates application; (v) once developed and characterised, aptamers can be produced in large quantities and at low cost; (vi) targeted modification, e.g., for programmable pharmacokinetics, is also possible. As a result, aptamers have great potential as receptors in detection systems and for therapeutic purposes [13,14].

The selection of nucleic acid-based aptamers was first described in 1990 by Tuerk and Gold, as well as by Ellington and Szostak, in two separate publications [1,15]. It follows the Darwinian evolutionary principle, consisting of sequence variation, selection, and replication and is, therefore, known as the ‘systematic evolution of ligands by exponential enrichment’ (SELEX) [15]. The starting point of this iterative process is a randomised sequence library with a diversity of ~10^15^ distinctive sequences [16]. Starting from this pool, the aptamers that bind a defined target with the highest affinity under specified conditions get selected by incubating the library with the target and washing off non-binding sequences. The bound aptamers are then eluted, amplified, and converted back into single-stranded oligonucleotides. The obtained subset of the original library represents the aptamer pool for the subsequent selection cycle. Selection takes place under increased selection pressure, which is in particular determined by the stringency of the washing step. After about five to twenty rounds, the remaining aptamer pool contains in the best case only a few, but highly affine aptamer sequences. This final aptamer pool is typically ligated into a vector, characterised by cloning and subsequent Sanger sequencing, and an extensive analysis of binding parameters is performed. Over the past three decades, many variations of this protocol have become established. What all SELEX methods have in common, however, is that the selection itself remains a ‘black box’—largely random and incomprehensible [17,18,19]. As a result, the success rate of SELEX is only about 30% [20,21].

To gain initial insights into the selection process and increase the success rate, SELEX can be combined with high-throughput sequencing (HiTS). SELEX linked with HiTS, was first described in 2002, but this method only became widely available with the introduction of commercial, next-generation sequencers, which is reflected in the increased number of publications from 2010 and onwards [22,23,24,25]. HiTS can be performed after each round of selection and enables a more comprehensive analysis of the obtained aptamer pools. This additional step allows the identification of functional and rare motifs, as well as the quantification of their abundance.

In parallel, high-throughput applications of biochemical microarrays have evolved significantly. The potential of applying this technology in an optically controlled selection of aptamers, by using a fluorescent target and measuring its binding to the immobilised DNA, was already recognised in 2004 by Asai et al. [26]. However, the aptamer sequences to be investigated have to be identified before the microarray is synthesised; hence, the pre-selection and sequencing of the obtained aptamer pool is necessary.

These two approaches were combined on a platform within an experiment called ‘high-throughput sequencing–fluorescent ligand interaction profiling’ (HiTS–FLIP). It was invented in 2011 by Nutiu et al. to study the binding of transcription factors to double-stranded DNA and is the first method that allows both high-throughput screening and quantitative measurement of the affinity of DNA-protein interactions [27]. HiTS–FLIP exploits the potential of Illumina sequencing flow cells to display an immense diversity of DNA sequences, on which—taking advantage of the sequencer’s optics—fluorescence-based affinity and specificity assays can be performed directly after sequencing. Thus, several million DNA sequences can be analysed in parallel and without bias for their affinity and specificity towards a fluorescent target at equilibrium [28]. This offers enormous potential for interaction studies. Thereby, the selection efficiency of affine sequences can be significantly increased, making the de novo discovery of aptamers orders of magnitude faster than that of any other affinity reagent [29]. In addition, this experiment allows for the answering of questions about the selection process and the relationship between the sequence, structure, and function of aptamers that were previously unanswerable.

Since its introduction, the HiTS–FLIP method has been modified and the scope of application greatly extended, mostly by performing additional steps between the HiTS and the FLIP. For example, it has been used to study ssDNA-cell [29] and RNA-protein [30,31], as well as peptide-protein interactions [32,33]. In addition, unnatural bases have been incorporated into DNA aptamers via click chemistry [28]. This review provides an overview of the HiTS–FLIP method and its variants, which have tremendous potential to revolutionise the selection of aptamers.

## 2. Description of the Method

### 2.1. Sequencing by Synthesis

At the beginning of a HiTS-FLIP experiment, a DNA library containing several million individual sequences with flanking adapters is bound to a flow cell via surface-bound oligos complementary to the library’s adapters. Immobilisation of the DNA sequences is followed by solid-phase bridge amplification, which generates clonal clusters less than one micron in diameter containing up to one thousand identical DNA molecules [34].

The sequence of all DNA clusters is then determined in a procedure called ‘sequencing by synthesis’. This method is based on the visualization of a repetitive incorporation of reversibly terminating, distinctly fluorescently labelled dNTPs by a DNA polymerase [34]. For this purpose, the flow cell is first incubated with 3′ blocked, fluorescently labelled nucleotides (a different fluorophore at each type of nucleotide) and sequencing primers that bind complementarily to the immobilized ssDNA. At each nucleic acid chain, the DNA polymerase extends the primers with one labelled dNTP and the unbound nucleotides get washed away. An image of the flow cell—showing the fluorescence of bound nucleotides—is then taken. Base calling is carried out for each cluster depending on the location, wavelength, and intensity of the detected fluorescence. Subsequently, the 3′ end group and the fluorophore of the bound nucleotides are cleaved off simultaneously, enabling the addition and detection of the next nucleotide. The described cycle is repeated 30 to 300 times for each read [35].

### 2.2. Ligand Interaction Profiling

**Figure 1:**
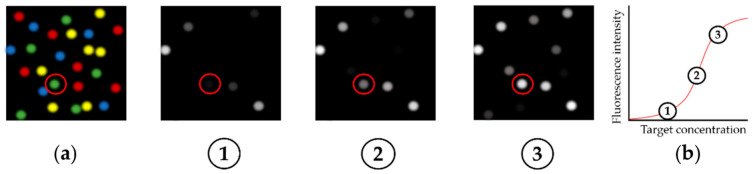
Schematic representation of the fluorescence signals detected in HiTS–FLIP and the resulting binding curve. First, sequencing by synthesis was carried out (**a**), whereby the positions of the clusters and their sequences were known. Subsequently, a fluorescence-labelled target was added to the flow cell in different concentrations and the fluorescence intensity was determined for each cluster, depending on the target concentration (**1**, **2**, and **3**). By fitting a sigmoidal function to this data, a binding curve could be generated for each cluster (exemplarily shown for the red-circled cluster, (**b**).

Analogous to fluorophore-labelled nucleotides, the optics of Illumina sequencing platforms can also detect the binding of other fluorescent ligands to the clusters displayed on the flow cell. This is exploited in ‘fluorescent ligand interaction profiling’ (FLIP), where a fluorescently-labelled target is added to the flow cell and a fluorescence image is taken after an equilibration period [27]. The fluorescence of each cluster can be quantified and aligned with the corresponding DNA sequence, based on its localisation on the flow cell (Figure 1). This direct observation of the binding of a target to DNA allows the unbiased determination of kinetic and thermodynamic binding parameters.

The equilibrium dissociation constant (*K*_d_) describes whether a complex is preferentially present in associated or dissociated form [36]. It can be measured by incubating the flow cell with different concentrations of the target and detecting the respective fluorescence signal after a sufficient equilibrium time [27]. The fluorescence intensity of each cluster can then be plotted as a function of the target concentration. This generates a binding curve—e.g., by fitting these data to a Hill-Langmuir function—from whose functional equation, *K*_d_ can be determined (compare Figure 1) [37,38]. Taking into account the temperature (*T*) and the molar gas constant (R), the standard Gibbs energy of formation of the bound aptamer/target complex ($\Delta_{r}G^{0}$) can then be determined according to Equation (1) [39].

(1) $$\Delta_{r}G^{0}=RT\;\cdot \;\ln\;Kd$$

The association rate constant (*k*_on_) and the dissociation rate constant (*k*_off_) describe the rate of complex formation and the stability of the complex. They can similarly be measured by sequentially imaging the fluorescence over time during binding of the target and during dilution of the bound fluorescently-labelled target on the flow cell [31].

### 2.3. Image Processing, as well as Kinetic and Thermodynamic Measurements

**Figure 2:**
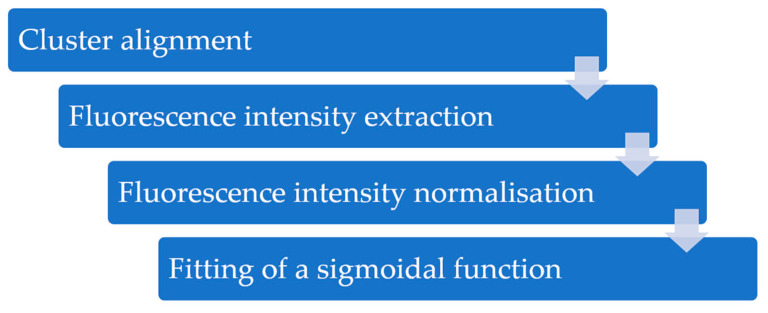
Overview of the image processing workflow. First, cluster centres obtained from sequencing were cross-correlated with the FLIP images, and the intensity from the registered fluorescent signals was extracted. Subsequently, the extracted fluorescence intensities were normalised, and the data aggregated across the images by cluster ID. Afterwards, a sigmoidal function was fitted to the aggregated data—the normalised fluorescence intensity, in dependence of target concentration—in order to obtain a binding curve and, hence, the *K*_d_ for each cluster.

In order to determine the fluorescence signal of the individual sequences, and thus, their *K*_d_, an alignment between the aptamer sequence and the fluorescence intensity profile of its clusters must be created over a range of target concentrations. For this purpose, processing of the FLIP images is necessary. A schematic workflow, containing the obligatory data processing steps, is shown in Figure 2.

The first step is to cross-correlate the positions of the clusters on the tiles of the flow cell derived from the FASTQ files, obtained from sequencing, with the fluorescent images of FLIP (so-called TIFF files). If the flow cell is not moved within the entire HiTS–FLIP experiment, this alignment is relatively simple [28,40]. However, if the platform is changed, i.e., from a sequencer to an imaging platform (compare Section 3.1), alignment markers (e.g., fluorescent oligonucleotides that are complementary to a constant sequence of the library) may be necessary for cross-correlation [41,42]. After alignment, the cluster centres must be defined, and their fluorescence intensity extracted from the FLIP images to determine the integrated fluorescence of each cluster. This can be achieved by fitting a sum of overlapping 2D Gaussian curves to the images, with each Gaussian curve centred at the position of each cluster [31]. The extracted intensities should then be normalised for each subtile. Cluster-related normalization factors can be obtained using information from the sequencing images, which can introduce a nucleotide-specific bias [27]. Alternatively, they can be determined from the target images, which more accurately captures the non-even illumination bias and can, therefore, lead to an improved correction for each tile image [40]. If the same sequence is present multiple times on the flow cell, the normalised fluorescence of those clusters can be averaged to minimise noise (compare Section 3.2) [31]. By correlating the normalised fluorescence intensity of each cluster or sequence with the target concentration and fitting a sigmoidal function to this aggregated data a binding curve for each sequence is obtained. From the binding curve, further parameters, such as *K*_d_, can be determined (compare Section 2.1). A ranking of the sequences, based on the fluorescence intensity can be carried out before or after formation of the binding curves with different methods, depending on the research question.

## 3. General Requirements and Considerations

### 3.1. Sequencing and Imaging Platforms

Various Illumina sequencers were used to perform HiTS–FLIP experiments. When choosing a platform, various aspects should be considered. On the one hand, the chosen platform determines the limitations of the aptamer display: the Illumina technology can efficiently cluster and analyse DNA sequences up to 1–2 kb long, with the maximum read length depending on the sequencing platform [32]. Currently, the MiSeq provides the longest reads, with up to 2 × 300 bases [35]. In addition, all microarrays have a limited capacity, due to the chips’ size and maximum cluster density. More specifically, a cluster density of 700–1400 clusters per square millimetre is recommended for unpatterned Illumina flow cells, while patterned flow cells enable the maintenance of high data quality, at even higher cluster densities [43]. The resulting capacity (in maximum number of reads per run) of current Illumina platforms is summarized in Table 1. Great molecular diversity of the starting libraries is very important to obtain aptamers with high affinity [16]. The simultaneous screening of as many as possible sequences is, therefore, favourable.

On the other hand, while the sequencers’ hardware is sufficient for HiTS–FLIP, the sequencers’ software has to be modified, in order to perform the experiment; such software modifications are neither intended nor supported by the manufacturer. Illumina sequencers are operated by extensible markup language (XML) recipes that encode the biochemical steps of the sequencing protocol—containing different commands to control the hardware. These commands can be used to encode the entire HiTS–FLIP protocol [27,28]. However, while the software was more accessible on the now obsolete Genome Analyzer IIx (GA IIx), changing the run files is more difficult on current sequencers. Therefore, after support for the GA IIx was discontinued by Illumina, mainly fluorescence microscopes or self-constructed platforms (often containing parts of the GA IIx) were used to visualize target-binding. Recently, Wu et al. managed to perform the HiTS–FLIP experiment completely automatically on a MiSeq, requiring only minor hardware modifications (i.e., an additional multiport and few tubing changes) and a set of custom software that will be freely available [28]. As a result, any laboratory with access to a MiSeq can now easily implement the HiTS–FLIP experiment. This is expected to significantly increase the popularity of HiTS–FLIP. The progression of the different platforms used within this experiment for HiTS and FLIP is summarized in Table 2. Remarkably, efforts regarding repurposing of other Illumina sequencers as fluorescence microscopes are also ongoing (e.g., of the HiSeq 2000 and 2500 in the so-called ReSeq project [50]).

Additionally, other parameters, i.e., the resolution and sensitivity of the optics, are also highly relevant for performing HiTS–FLIP experiments. For example, the GA IIx had total internal reflection fluorescence (TIRF)-based optics that created an evanescent wave that extended only up to 200 nm into the flow cell [34]. Contrary, newer sequencing platforms feature different optics, e.g., a line-scanning confocal imaging apparatus that has a depth-resolution of ~500 nm [57]. Therefore, with newer sequencing platforms, washing away unbound fluorophores before imaging the fluorescence may be necessary.

### 3.2. Libraries

**Figure 3:**
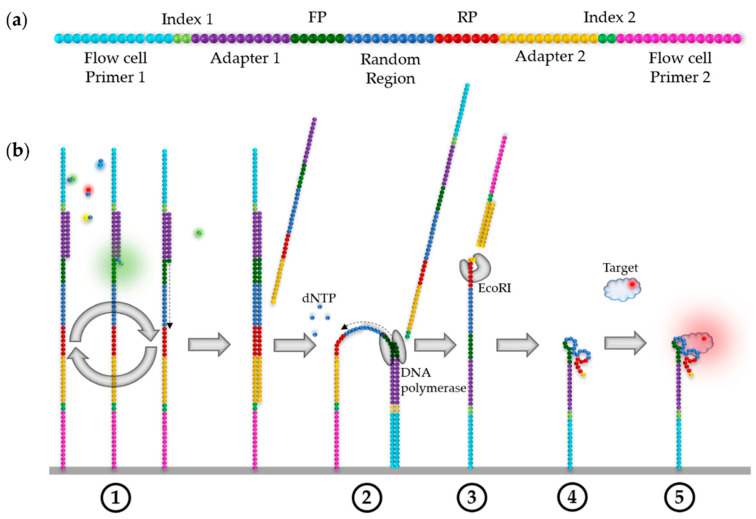
Schematic illustration of the HiTS–FLIP experiment for ssDNA aptamers, according to Wu et al. [28]. (**a**) The initial DNA library, designed for paired-end sequencing. A restriction enzyme (EcoRI) recognition sequence is inserted between the reverse primer and adapter 2. One dot generally corresponds to ~3 nt; however, the possible length of the randomised core is highly variable, ranging from ~10–600 nt; FP: forward primer; RP: reverse primer. (**b**) The HiTS–FLIP process for ssDNA; dNTP: Deoxyribonucleotide triphosphate.

The DNA libraries used for HiTS–FLIP contain a randomized central core, flanked by constant sequences required for binding to the flow cell, as well as amplification and sequencing, as shown in Figure 3a. Various parameters, including the length of the randomised region, should be considered in the design of a DNA library because they can greatly influence the outcome of a selection experiment [16].

There are three main approaches to design the variable sequence:

1 A fully random sequence. This type of library is especially suitable for the de novo discovery of aptamers. It must be kept in mind that even the NovaSeq can only display about one hundred thousandth of a random DNA library—typically containing ~1 × 10^15^ different molecules (compare Table 1) [16]. However, each sequence should be represented, on average, by at least ten clusters on the flow cell, in order to reduce measurement noise and possible bias, by performing several distributed measurements for each sequence (compare Section 2.3) [51,58]. Hence, it can be necessary to reduce fully random libraries for HiTS–FLIP to a diversity of ~10^6^ different sequences, e.g., by performing a few rounds of conventional SELEX [28,29]. This allows all sequences to be efficiently displayed simultaneously on the flow cell.
2 A natural (e.g., genomic/transcriptomic) library. This method takes advantage of the large structural and functional diversity of nucleic acid sequences inherent to biological systems. Analogous to the workflow of genome sequencing, the genome of an organism is fragmented, and these fragments (<300 bp) are used as a library. This type of library can be used to study the binding of RNA binding proteins (RBPs) to the transcriptome, as applied in the ‘transcribed genome array’ (TGA)—a RNA HiTS–FLIP variant developed by She et al. [52].
3 A partially random (doped/mutant) library based on a known consensus sequence motif or a known aptamer. For this purpose, single, double, and even higher-order mutations can be introduced into the known sequence. They can either be generated randomly, e.g., using the error-prone polymerase chain reaction (PCR) [30] and by degenerated oligo synthesis [31], or in a programmed manner by array-based synthesis [59,60,61]. The latter enables almost equimolar synthesis of up to 10^6^ designed molecules. This kind of library is especially feasible for analysing and optimising already selected aptamers in mutational assays (see Section 5).

The variable sequence—the actual aptamer—must be flanked with constant sequences for the HiTS–FLIP process. If a random library (1) is used, first primer sequences (~20 nt) must be added to amplify the library during the SELEX process. Illumina-defined adapter sequences (~35 nt) are ligated to these, which act as primer sequences for sequencing by synthesis. The adapters are followed by indices (~8 nt) that serve to distinguish the sequenced samples. Finally, flow cell primers (~25 nt) must be ligated, which hybridise complementarily to the oligonucleotides bound on the flow cell, and thus, enable the immobilisation of the library on the flow cell. They also serve as primer sequences for bridge amplification [34].

In order for the different oligonucleotide clusters to be well distinguished by the instrument, a sufficiently high base diversity must be ensured during the first four cycles of sequencing [62]. Hence, either a high-diversity library (such as PhiX) should be spiked in or a short random region (e.g., ≥4 nt) could be included between the forward primer and the sequencing primers [28].

When selecting aptamers that are covalently bound to the flow cell via HiTS–FLIP, the fixed regions (adapter, index, and flow cell primer) that are not bound to the flow cell can be cleaved off during the experiment to prevent potential steric hindrance within the FLIP, as well as interactions of these sequences with the variable region. For this purpose, the recognition sequence of a restriction enzyme (e.g., EcoRI) must be inserted between the respective primer and adapter during the preparation of the DNA library. Alternatively (or additionally), oligonucleotides complementary to the constant regions of the library can be added to the flow cell to stabilize the desired aptamer structure [63].

### 3.3. Targets

The most important prerequisite for performing a HiTS–FLIP experiment is that the target either fluoresces itself or can be fluorescently-labelled. Furthermore, the fluorescence must match the excitation and detection wavelengths of the optics of the sequencer/FLIP platform used. An overview of the excitation wavelengths of the GA IIx and current Illumina sequencing platforms is, therefore, provided in Table S1. The emission wavelengths are not published by Illumina. However, according to their 2007 patent application on dye compounds and the use of their labelled conjugates (US Patent Nr. 8178360), the fluorescent nucleotides used in four channel detection include 9-(2-Carboxyphenyl)xanthylium dye ‘Dye 2′-dTTP, Atto532-dGTP, DY681-dCTP, and Alexa 647-dATP [64]. Hence, fluorophores, whose fluorescence spectra are similar to those mentioned above, are suitable for a HiTS–FLIP experiment on four-channel sequencers. Exemplary fluorophores used for HiTS–FLIP experiments are listed, with their characteristics, in Table S2 [65,66,67,68,69,70,71,72].

Another factor to be considered in HiTS–FLIP is the size of the target: On the one hand, fluorescence labelling depends on the presence of certain functional groups and can be difficult for small molecules without significantly affecting their molecular properties [73]. However, targets are often immobilised in SELEX experiments, e.g., bound to beads [74]. The same chemistry can be exploited for fluorescent labelling in many cases. Furthermore, different riboswitches, combined from two aptamers—one for a non-fluorescent small molecule (‘sensor’) and one for a fluorogenic molecule (‘reporter’)—were already analysed using HiTS–FLIP [56]. It would be conceivable to assay aptamers against non-fluorescent small molecules, via such an existing riboswitch system, if the corresponding aptamer part of the riboswitch is mutated. On the other hand, for larger targets, such as eucaryotic cells, it is very likely that several clusters lay underneath each target, making the assignment of affine sequences more difficult [29].

Because of the aspects outlined, HiTS–FLIP is particularly easily applicable but not limited to investigating interactions with proteins. For example, HiTS–FLIP experiments have been successfully performed with transcription factors [27,40], RNA-binding proteins [30,51,52,53], Cas9 and Cas3 [41,42], antibodies [32,33], and other proteins [28,30,31,32,33,51], as well as with the fluorogenic small molecules DFHBI [33] and malachite green [56], the peptide insulin [28], and RNA [54,55]. Mamet et al. even demonstrated that the affinity of particular sequences towards human tumour cells can be determined via HiTS–FLIP, based on their ‘bound fraction’—the ratio of the number of times one of the sequences’ clusters co-localized with a bound cell to the total number of clusters of that particular sequence [29]. Hence, targets with a diameter greater than 10 µm can also be analysed using HiTS–FLIP if the sequences are displayed multiple times on the flow cell. This can easily be achieved by using a library with lower diversity. This underlines that the range of possible targets for HiTS–FLIP is almost as large as for SELEX. An overview of the aptamers that have already been selected and optimised by HiTS–FLIP is provided in Table S3.

## 4. Aptamer Selection Methods

### 4.1. DNA Aptamers

The simplest, and probably most obvious, application of HiTS–FLIP is the analysis of DNA-protein interactions. In its original form, the HiTS–FLIP experiment was used to analyse the binding sites of transcription factors to dsDNA [27]. By now it has additionally been utilised to assay ssDNA-protein [28] and ssDNA-cell interactions [29].

The outline of the ssDNA HiTS–FLIP experiment, according to Wu et al. [28], is shown in Figure 3b. Although it would be conceivable to carry out the FLIP experiment in a single-end run—directly after sequencing—when analysing ssDNA sequences, paired-end sequencing can also be performed. In paired-end sequencing, an antisense DNA library is bound to the flow cell, then amplified and sequenced (1), offering the advantages that the generated FASTQ file contains the sense sequence of the clusters and that the sense strands can be resynthesized in the paired-end turnaround using unmodified nucleotides, without changes in the sequencing protocol (2). The resynthesis is necessary because the strands synthesized during sequencing contain modified bases. After sequencing, adapter 2, index 2, and flow cell primer 2 can be cleaved off to prevent potential steric hindrance within the FLIP, as well as interactions of these sequences with the random region (3). For this purpose, the recognition sequence of a restriction enzyme (e.g., EcoRI) must be inserted between the reverse primer (RP) and adapter 2 during the preparation of the DNA library. By incubating the flow cell with oligonucleotides complementary to the recognition and adapter 2 sequence, a double-stranded cut site is formed, enabling cleavage with the corresponding restriction enzyme. Alternatively, oligonucleotides complementary to the constant regions and flanking the variable core of the library can be added to the flow cell to block interferences of those regions with the 3D structure of the folded aptamer [29,63]. Correct folding of the aptamers can be ensured by heating the flow cell to 95 °C, cooling at 0 °C, and afterwards incubating at the selection temperature, e.g., 37 °C, for 10 min each (4) [29]. After a washing step, FLIP is performed by incubating the flow cell with different concentrations of fluorescently-labelled target, washing away unbound target molecules and subsequently imaging the flow cell (5).

For example, Mamet et al. used HiTS–FLIP to select affine DNA aptamers that induce apoptosis of various primary human tumour cells within only 3 h, as detected by a pre-loaded fluorogenic reporter of apoptosis [29]. Due to the rapid ab initio discovery of new potential drugs, the HiTS–FLIP experiment could be a promising tool for the development of personalised medicine.

### 4.2. Base-Modified DNA Aptamers

A major limitation of nucleic acid-based aptamers is their hydrophilic nature, which restricts the potential hydrophobic interactions with target molecules. The inclusion of base-modified, non-natural nucleotides can, therefore, increase not only the structural but also the chemical diversity of aptamer pools and, thus, provide improved target recognition functionality [75,76,77,78,79]. One way to efficiently functionalise nucleic acids, prior to selection, is the click-SELEX method, established in 2015, by Tolle et al. [80]. In this method, the deoxythymidines in the random region of a DNA library are replaced by alkyne-modified dUTPs that are recognised by type B polymerases and, thus, enable the amplification of the modified oligonucleotides [81]. The alkyne function can be further derivatised by a copper (I)-catalysed azide-alkyne cycloaddition (CuAAC), allowing nucleic acids to be modified with a variety of organic azides [80,82].

This click-chemistry was recently implemented in HiTS–FLIP by Wu et al. [28]. Their so-called ‘non-natural aptamer array’ (N2A2) represents the first automated system that enables screening of base-modified aptamers. The workflow of N2A2 is similar to that described for DNA aptamers in Section 4.1 but includes an intermediate step between HiTS and FLIP, the conversion of the aptamers, which is performed during paired-end sequencing.

The workflow of N2A2 begins analogously with sequencing an antisense DNA library identical to that described above. During the paired-end turnaround step (step 2 in Figure 3b), C8-alkyne-dUTP is replaced by dTTP and incorporated using the KOD-XL polymerase. Hence, alkyne functions are inserted at each ‘thymine-position’, which are subsequently modified with organic azides via click chemistry. To validate the successful C8 alkyne-dUTP incorporation, the synthesis of a fiducial mark sequence is controlled. For this purpose, in addition to the DNA library, a fiducial-mark sequence (containing adenines) is modified with adapters, indices, and flow cell primers and immobilised on the flow cell. After paired-end turnaround, the flow cell is incubated with fluorophore-labelled strands complementary to the fiducial mark, so that fluorescence is only visible if the paired end turnaround was successful. As described in Section 4.1, Wu et al. had subsequently removed the adapter and flow cell primer sequences attached to the 3′ end of the aptamers by enzymatic digestion, to prevent interactions of these sequences with the random region and potential steric hindrance. The organic azides are then conjugated to the aptamers by click chemistry during the second read of the paired-end sequencing process. The click-reaction procedure on the flow cell can be validated by introducing an azide-labelled fluorophore (e.g., Cy3) [28].

Wu et al. impressively demonstrated that this version of the HiTS–FLIP experiment can be used to efficiently select multiple, base-modified aptamers with low nanomolar affinity and very high specificity towards their target [28]. First, they investigated the effects of two different modifications, the amino acids tyrosine (Y) and tryptophan (W), in place of thymine, on the affinity of DNA aptamers for VEGF. For this purpose, they performed three separate HiTS–FLIP experiments with a pre-enriched DNA library, two with base-modified (Y or W) and one with natural DNA aptamers. Especially, the tryptophan-modified aptamers exhibited a considerably higher affinity for VEGF than the natural DNA aptamers. In particular, they were able to select an aptamer (V4) with a calculated *K*_d_ of (2.8 ± 0.6) nM, whereas the previously known DNA aptamer SL_2_-B [83] exhibited a *K*_d_ of only 18.7 nM in the same assay. Second, the tryptophan-modified aptamer fet4 was developed, which can discriminate between two glycoforms of one protein—it binds fetuin (*K*_d_ ~3 μM) but not asilianofetuin. Third, ins24 was selected, a phenylalanine-modified aptamer, which binds specifically and affinely to insulin, even in diluted human serum (*K*_d_ = 4.8 μM). In comparison, the previously known aptamer IGA3 [84] showed no measurable affinity towards insulin in this complex matrix [28].

### 4.3. RNA Aptamers

The relationship between the sequence, structure, and function of RNA is extremely complex [85,86,87]. High-throughput methods that can quantitatively measure macromolecular interactions of RNA are, therefore, of great interest. The highly related HiTS–FLIP variants, entitled ‘high-throughput sequencing and RNA affinity profiling’ (HiTS–RAP) [30] and ‘quantitative analysis of RNA on a massively parallel array’ (RNA–MaP) [31], represent arrays that enable the equally comprehensive and quantitative characterization of binding to RNA. These two methods and their applications have already been reviewed in detail elsewhere [58].

**Figure 4:**
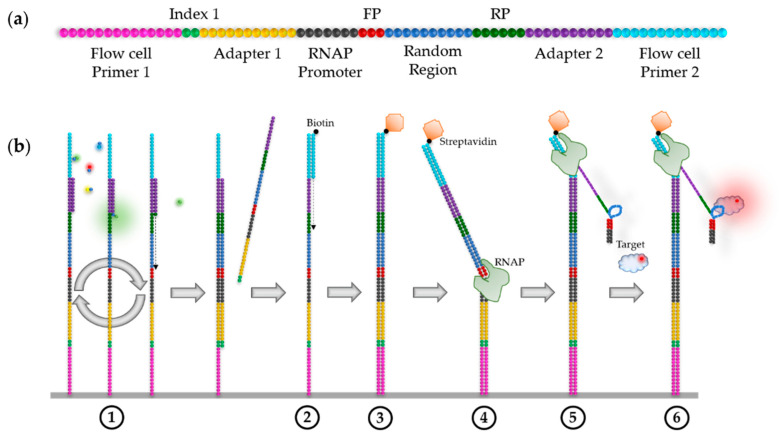
Schematic illustration of the RNA–MaP experiment, according to Buenrostro et al. [31]. (**a**) The initial DNA library designed for single-end sequencing, similar to the one used for DNA aptamer selection (Section 4.1), but with a RNA polymerase (RNAP) promotor at the 5′ end of the aptamer sequence. (**b**) The RNA–MaP process.

For these two HiTS–FLIP variants, which require in vitro transcription of the DNA clusters, an RNA polymerase (RNAP) promoter must be added in the initial library, to the 5′ end of the variable region (see Figure 4a). The termination site of the RNAP and the 3′ end of the variable sequence should be separated by at least 25 nt—the approximate length of the RNAP exit tunnel [88].

To analyse RNA via HiTS–FLIP, the DNA required for sequencing by synthesis must be converted into RNA directly on the flow cell, prior to the FLIP. This in situ transcription of the DNA can be performed in three steps, as shown in Figure 4b. After sequencing (1), the ssDNA is converted into dsDNA by annealing primers and extension by a DNA polymerase, e.g., the Klenow enzyme (2) [51]. Then, a steric blockade must be added to the 5′ ends of the complementary aptamer sequence. There are two distinct approaches for this: RNA–MaP uses biotinylated primers for the resynthesis of dsDNA. The biotin can subsequently be bound by streptavidin (3) [31]. In HiTS–RAP, the DNA library preparation involves the insertion of a 32 bp *Ter* consensus element at the 3′ end of the transcribed region, between the aptamer and primer sequences, which is bound by the *Escherichia coli* replication terminator protein Tus [30,89]. The flow cell is, afterwards, incubated with an RNA polymerase (e.g., T7 or *E. coli* RNAP) that binds to the dsDNA in a sequence-specific manner and starts transcription (4). To ensure that only one transcript per DNA template is produced, the RNAP can be reversibly stalled on the DNA after transcription of about 30 nt—the length of its footprint [31]. For this, the first 30 nt after the promotor must be composed of only three bases (e.g., only A, C, and T), followed by the fourth base (e.g., G). Additionally, transcription must be initiated under starvation conditions—i.e., lacking the nucleotide complementary to the stall base (e.g., CTP). Therefore, the RNAP stops at the first position, where the missing nucleotide should be included—the stall base. If the distance from the stall base to the promoter equals the footprint of the RNAP, the RNAP stops stably on the DNA template and binding of another RNAP is prohibited [88]. After washing away excess RNAP, transcription can be resumed by adding the missing nucleotide to the flow cell. Transcription continues until the RNAP reaches the steric blockade (Tus or streptavidin), which forces the RNA polymerase to stall on the DNA template [88]. As a result, the nascent RNA transcript is stably displayed on the flow cell (5). Transcription efficiency can be determined by hybridising fluorescently-labelled DNA oligonucleotides to the constant sequences of the transcripts [31]. This also provides a mark that can be used for cluster alignment (compare Section 2.3). Furthermore, analogous to DNA aptamers (see Section 4.1) annealing complementary oligos to the constant regions of a library, promotes the desired independent folding of the variable region [63]. FLIP is then performed by adding different concentrations of the fluorescently-labelled target to the flow cell and detecting the fluorescence of the clusters (6).

The ‘transcribed genome array’ (TGA), developed by She et al., is a refined version of the RNA–MaP approach that utilizes the genome of an organism as the random region of the library (see Section 3.2) [52]. TGA allows the analysis of the binding sites of RBPs within a whole transcriptome, at single-base resolution, in a single experiment. As an example, the binding of Vts1 to the transcriptome of *Saccharomyces cerevisiae* was investigated. This provided similar results to those obtained from in vivo experiments but with deeper insight in the binding sites, revealing a highly specific sequence and structure-binding motif [52].

**Figure 5:**
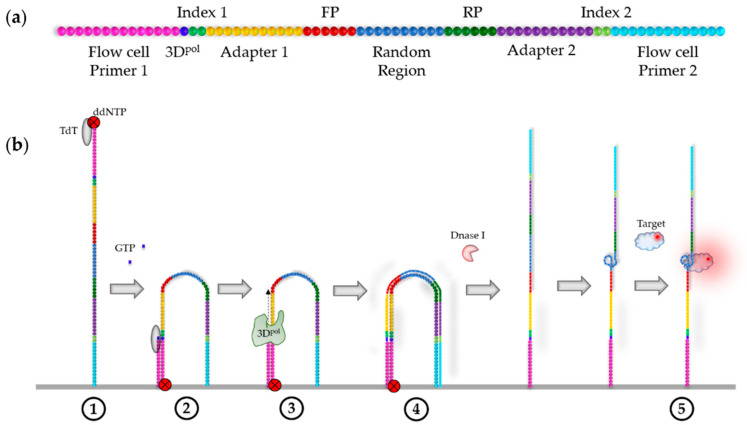
Illustration of HiTS–FLIP with RNA clusters covalently linked to the flow cell, according to Svensen et al. [33]. (**a**) The initial DNA library, designed for paired-end sequencing; 3D^pol^: initiation site for poliovirus RNAP; FP: forward primer; RP: reverse primer. (**b**) The RNA HiTS–FLIP process, starting from the sequenced DNA strands; TdT: terminal deoxyribonucleotidyl transferase; 3D^pol^: poliovirus RNAP; GTP: guanosine triphosphate.

Alternatively, RNA can be synthesised covalently bound to the flow cell by a primer-dependent RNAP. This enables a higher stability of the displayed RNA (>72 h), as shown in Figure 5b [33]. While the RNAPs that use DNA primers have not yet been described, RNAPs dependent on RNA primers are known. For example, the poliovirus 3D polymerase (3D^pol^) can transcribe DNA templates in vitro by extending the 3′-OH of an RNA primer [90,91,92,93]. Therefore, flow cell primers must be modified after sequencing with 3′ ribonucleotides to be applicable for priming transcription via 3D^pol^, i.e., by using the terminal deoxyribonucleotidyl transferase (TdT) [94]. At first, the 3′ ends of the DNA clusters can be blocked (1), e.g., by labelling with FITC-ddATP using TdT [33]. Importantly, the flow cell primers are 3′ phosphorylated and, hence, not modified in this step. After the 3′ ends of the DNA clusters are blocked, the flow cell primers are dephosphorylated using the polynucleotide kinase. TdT and GTP are then applied to the flow cell, resulting in the addition of 2–3 guanosine ribonucleotides at the 3′ end of the flow cell primers (2), which bind complementarily to three cytosines, incorporated in the DNA library as the first residues at the 3′ of the flow cell primers (3D^pol^ in Figure 5a). Subsequently, transcription occurs by incubating the flow cell with 3D^pol^ and ribonucleotides (3). After cRNA synthesis, the DNA strands in the DNA/RNA duplexes are selectively degraded using DNase I, resulting in ssRNA clusters that are covalently linked to the flow cell (4). DNase I has a preference for pyrimidine-purine-pyrimidine sequences; thus, degradation of the DNA in the primer of the RNA strands can be minimized by using flow cell primers that contain this sequence motif as rarely as possible [95]. In particular, Svensen et al. showed that DNase I treatment did not reduce the amount of RNA strands with a DNA primer domain, while ~60% of the DNA strands in the clusters were digested [33]. Analogous to RNA–MaP and HiTS–RAP, successful transcription can be confirmed by incubating the flow cell with fluorescently-labelled oligonucleotides complementary to the 3′ end of the new synthesized RNA and detecting the clusters’ fluorescence. After correct folding of the aptamers, FLIP can be performed (5).

### 4.4. Peptide Aptamers

HiTS–FLIP can also be used for directly assaying the influence of peptide or protein sequence variations on their function, as demonstrated by Svensen et al. [33], and exploited in the so-called ‘protein display on a massively parallel array’ (Prot–MaP) by Layton et al. [32]. To synthesize peptides encoded by DNA clusters, the DNA clusters must first be converted into RNA clusters, which then serve as templates for in vitro translation. For the stability of the peptide display, it is preferable to synthesise the RNA covalently bound to the flow cell [33], while translation of RNA stably bound to a RNAP is also possible [32]. Transcription can, therefore, be performed with either method described in Section 4.3.

**Figure 6:**
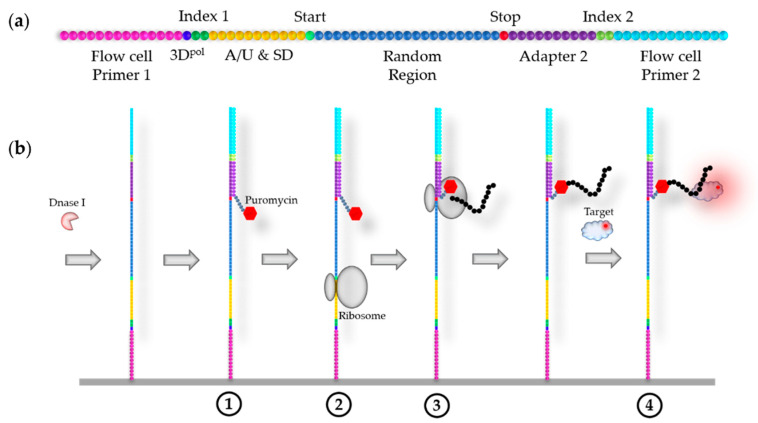
Illustration of peptide display HiTS–FLIP, according to Svensen et al. [33]. (**a**) The initial DNA library designed for paired-end sequencing, followed by in situ transcription and translation; 3D^pol^: initiation site for poliovirus RNAP; A/U: A/U-rich translation initiation enhancer; SD: Shine–Dalgarno sequence; Start: start codon; Stop: stop codon. (**b**) The peptide display HiTS–FLIP process starting with the RNA clusters, generated as depicted in Figure 5b.

For translation and peptide display, ancillary features have to be included in the DNA library. To initiate translation, in addition to features required for transcription, the 5′ adapter sequence must also contain a ribosome binding site (Shine–Dalgarno sequence [96]) and preferably an upstream A/U-rich translation initiation enhancer [97], followed by a start codon. Two different approaches have been described for the termination of the translation, which allow for a stable display of the peptides on the flow cell. In Prot–MaP, the ribosome is stably and efficiently stalled on the RNA after the aptamer sequence has been translated [32]. For this purpose, a ribosome stall sequence containing a polyproline (PPP) motif is integrated in the 3′ adapter, which causes effective translational pausing in the absence of elongation factor P (EF-P) [98,99]. Additionally, critical residues of SecM and TnaC, that presumably promote stalling via direct interactions with the exit tunnel, can be integrated upstream of the PPP-motif [32,100,101]. Alternatively, for a ribosome-free display, the nascent peptide chain can be transferred to RNA-bound puromycin after stalling the ribosome at the stop codon (Figure 6b) [33]. Hence, the 3′ adaptor should feature a ribosome stall sequence and/or a stop codon (Figure 6a and Figure S1a). In addition, it is necessary to include flexible spacer sequences at the C-terminal end of the translated region to allow accessibility and independent folding of the random peptide [102]. If a ribosome display is used, this linker should be at least as long as the ribosome’s exit tunnel; however, the efficiency of the display can increase with further spacer length [103].

The process of HiTS–FLIP on a ribosome-free peptide display, according to Svensen et al. [33], is shown in Figure 6b and described as follows. After sequencing by synthesis, the DNA clusters are converted into RNA clusters, and the DNA strands of the DNA/RNA duplexes are digested by DNase I (as described in Section 4.3, Figure 5b). Complementary oligonucleotide sequences to the constant 3′ end of the RNAs, labelled with puromycin via a flexible linker, are then hybridised to the clusters (1). Subsequently, bacterial ribosomes are injected to the flow cell, which bind to the Shine–Dalgarno sequence and initiate translation of the RNAs at the start codon (2) [96]. Translation continues until the ribosome encounters the stop codon and puromycin (3). Puromycin is a translation terminator because it is structurally similar to a tyrosyl-tRNA; therefore, the nascent peptide chain is transferred to puromycin during peptidyl transfer when it binds to the A site of the ribosome [104]. This forms a peptide-RNA conjugate that prevents further elongation, and the ribosome is released [105,106]. Afterwards, FLIP can be performed (4).

In Prot–MaP, transcription is performed, according to the RNA–MaP protocol (see Figure 4b) [32]. In vitro translation is then carried out using bacterial ribosomes, without any intermediate steps and in the absence of EF-P and release factor 1 (RF-1), ensuring stable and efficient stalling of the ribosome at the PPP-motif (shown in Figure S1). After ribosome stalling, FLIP can be conducted on this array.

It has been shown that the displayed peptides (e.g., FLAG peptides), obtained with both methods, can be bound by cognate antibodies, confirming that the peptides are accessible to proteins in solution [32,33]. Hence, HiTS–FLIP can be used analogously to a massive, multiplexed ELISA.

## 5. Mutational Assays

The structure of an aptamer is crucial for the affinity and specificity of its interactions with the target. Therefore, an in-depth understanding of the complex interactions involved between aptamers and their targets could be used to optimise the aptamers’ structure for their intended application [107]. However, most techniques applied for characterisation of aptamer binding sites, e.g., extensive truncation and mutant assays, are very laborious and time consuming [83,108]. Furthermore, crystal structures of aptamer-protein complexes, although providing very detailed information, have been identified only to a limited extent, as the crystallization of these complexes can be very challenging [109].

HiTS–FLIP offers a highly efficient alternative to conventional mutagenesis assays for a broad variety of natural and base-modified aptamers. It allows for the simultaneous screening of every possible single- and double-mutant variant of an aptamer, regarding its function, providing access to comprehensive functional sequence fitness landscapes like no other technique. Based on the heatmap, which depicts the change in affinity as a function of mutation, known aptamers can be rapidly optimised by identifying mutations that contribute to an increase in binding (compare Table S3).

In addition, combining the heatmap of normalised fluorescence intensity values for each mutant with secondary structure prediction for canonical base pairing provides great insight into how an aptamer interacts with its target. More specifically, the structurally and functionally important features that determine these interactions can be identified based on their impact on affinity—their sensitivity to mutation. Furthermore, the relative contributions of primary and secondary structure changes to binding energy, and even context dependence of preference for intermediates in secondary structure (e.g., G:U over C:A), can be investigated [31]. For example, by analysing the binding of the MS2 protein to mutants of its RNA recognition motif via RNA–MaP, Buenrostro et al. showed that the kinetic drivers of the observed affinity changes of RNA aptamers seem to be position-specific and often act by modulating association rates, probably by altering hairpin stability [31]. It is also possible to observe non-additive effects of mutations—epistasis and cooperativity [31,32]. Overall, the information gained from the HiTS–FLIP mutational assays create a valuable framework for detailed understanding of the sequence-specific rules driving acquisition of affinity in the selection process and, hence, for the evolution of new aptamers.

To demonstrate the enormous potential of HiTS–FLIP for mutation assays, Wu et al. examined the entire affinity landscape of all single- and double-nucleotide mutants of ins24 (compare Section 4.2) in a single HiTS–FLIP run [28]. They were able to identify bases and secondary structure elements that are significant for insulin binding and selected a double mutant that showed ~60% improvement in affinity [28]. Jarmoskaite et al. determined the sequence preferences of the human RBPs PUM1 and PUM2 by screening the binding to a mutant library of the known consensus motif with RNA–MaP [53]. This revealed a complex binding process with dependencies on the secondary structure, binding to ‘flipped-out’ residues, as well as energetic coupling [53]. Furthermore, using HiTS–RAP, Tome et al. characterised the mutants of two previously known aptamers targeting *Drosophila* NELF-E (Napt1min [110]) and GFP (AP3 [111]) [30]. While the effects of multiple mutations were mostly additive in the NELF aptamer, significant epistasis was observed in the GFP aptamer. Additionally, a GFP aptamer that conferred several-fold higher affinity than the previously known aptamer was obtained [30].

## 6. Conclusions

HiTS–FLIP is a very promising technique for the selection of a wide range of high-performance aptamers. It allows for simultaneous screening for the affinity and specificity of millions to billions of aptamers in a single experiment, greatly accelerating and simplifying access to these powerful and promising affinity reagents. While the selection of aptamers using SELEX often takes several weeks and has a success rate of merely 30% [20,21], aptamers can be selected in a completely automated manner within a few days or even hours using HiTS–FLIP [28]. Therefore, HiTS–FLIP enables to efficiently conduct a ‘per-target tailoring approach’, which can, for example, boost the development of patient-centred medicine for individual treatment of complex diseases, such as cancer [29]. In the HiTS–SELEX methods, which allow a similar high-throughput as HiTS–FLIP, affinity is indirectly obtained from the quantitative ratios of the sequences in the selected aptamer pools, so that a relatively high sequencing depth is necessary to generate the required information. Moreover, the redundancy of aptamer sequences in a pool is often biased by amplification efficiency [112]. In HiTS–FLIP, on the contrary, each cluster (i.e., each read) generates direct and independent information regarding its affinity and specificity towards a target. Furthermore, the exceptional direct and quantitative measurements of the linkage between sequence and function provides a deeper understanding of the interactions between affinity reagents and their targets, while eliminating complexities, such as amplification bias and stochastic dropout [32]. Compared to microarrays, HiTS–FLIP allows for the screening of significantly more and longer sequences, in parallel. Prot–MaP, for example, enables the display of proteins with a length of up to 200 amino acids, whereas peptide array platforms are typically limited to about 16 amino acids [32]. Therefore, HiTS–FLIP is suitable to analyse even complex structures, such as ribozymes and riboswitches [56], making HiTS–FLIP a more versatile technology. In addition, depending on the aptamers analysed, HiTS–FLIP can be more cost-effective than microarrays, since the cost of this technology is similar to that of regular Illumina sequencing [32].

The limitations of the HiTS–FLIP technology are mainly due to the sequencing platform used. For example, a maximum of 1–2 kb long DNA sequences can be efficiently clustered and sequenced, while the read length is limited to 2 × 300 bases (when using a MiSeq). In addition, the DNA sequencing error rate of Illumina sequencing, of ~0.1%, which gradually increases towards the end of the reads, can bias the HiTS–FLIP experiment, especially when very similar sequences are analysed, such as in mutation assays (Section 5) [113]. Since each sequence should ideally be displayed several times on the flow cell for a HiTS–FLIP experiment, sequence identifiers, in the form of indices or barcodes, can be added to the bottlenecked sequences prior to amplification and subsequent loading onto the flow cell. This allows for the identification of missequenced clusters, by comparison with the identifiers and sequences of the other clusters [31]. These steps are routinely included in the Illumina assay, and thus, do not represent additional effort. In addition, the use of paired-end sequencing can further minimise sequencing errors. Despite the good correlation between the binding affinities measured by HiTS–FLIP and other methods, the aptamers displayed in HiTS–FLIP can be susceptible to steric hindrance effects, especially for large targets. Therefore, variations in binding can be observed at different clusters with the same sequence [51]. This limitation can be overcome by averaging the fluorescence intensities of each target concentration for all clusters with identical sequence prior to estimation of *K*_d_ (see Section 2.3). Another challenge may arise from the photostability of the fluorophore used—if a highly dynamic process is monitored, differential photobleaching can bias the HiTS–FLIP experiment [41].

Although the application of HiTS–FLIP was initially limited to DNA/protein interactions [27], a variety of modifications of the experimental protocol have by now been described, allowing the affinity and specificity of base-modified DNA, RNA, peptides, and even proteins to be investigated. The range of possible targets has also been expanded—reaching from small molecules [33] up to human cells [29].

In addition, the performance of the HiTS–FLIP experiment was previously significantly limited by its reliance on the Illumina Genome Analyzer sequencing platform, which is no longer state of the art [27]. With the recent implementation of a fully automated HiTS–FLIP experiment on a MiSeq—a commonly used sequencer—and the publication of the required software and minor hardware modifications [28], it is now possible to easily perform the HiTS–FLIP technique in any laboratory with access to a MiSeq. Additionally, further efforts to implement HiTS–FLIP on other instruments are underway [50]. It can, therefore, be assumed that the HiTS–FLIP experiment will gain in importance in the future.

Due to its versatility, the HiTS–FLIP experiment can be applied in many other research areas. For example, the affinity and specificity of the CRISPR-associates protein 9 (Cas9) and type I-E CRISPR-Cas (Cascade) complex, as well as CRISPR-associates protein 3 (Cas3), were investigated by measuring the effects of combinatorial mismatches between guide RNA and target nucleotides. This made it possible to obtain comprehensive profiles of the specificity of the protospacer adjacent motif (PAM), as well as of the off-target binding behaviour in vitro [41,42]. Moreover, RNA–MaP was used to determine the sequence dependence and thermodynamic stability of the formation of various RNA junctions and helices, using the tectoRNA system [54,55]. In addition, Prot–MaP was used to select a ‘super-FLAG’ peptide sequence that has an almost eightfold lower limit of detection than the FLAG peptide. Furthermore, the relationship between protein sequence variation and catalysis of the O^6^-alkylguanine-DNA alkyltransferase was screened, demonstrating the potential of HiTS–FLIP to provide detailed information on the cooperativity and position-specific required backbone flexibility of full-length functional proteins [32]. Hence, HiTS–FLIP can greatly expand the understanding of the functional impact of coding mutations and amino acid interaction networks, providing a basis for the rational design of protein function [32]. The multicolour imaging capabilities of Illumina sequencers can even enable the measurement of complex biological interactions between differentially labelled binding partners and the implementation of other fluorescently measurable assays (e.g., for conformational changes) as the fluorescence resonance energy transfer (FRET) [114].

## Figures and Tables

**Table 1:** Comparison of the maximum number of single reads per flow cell and run time of the Genome Analyzer IIx (GA IIx) and current Illumina sequencing platforms, according to the manufacturer’s specifications.

Sequencing Platform	Maximum Reads Per Run	Run Time	Reference
GA IIx	168 million	2.5–9.5 day	[44]
iSeq 100	4 million	9.5–19 h	[45]
MiniSeq	25 million	4–24 h	[46]
MiSeq	25 million	4–55 h	[35]
NextSeq 550	400 million	12–30 h	[47]
NextSeq 1000/2000	1.1 billion	11–48 h	[48]
NovaSeq 6000	20 billion	13–44 h	[49]

**Table 2:** Evolution of the hardware used for HiTS–FLIP.

Publication Year	HiTS Platform	FLIP Platform	References
2011–2017	GA IIx	GA IIx	[27,30,31,40,41,51]
2016	GA IIx	Epifluorescence microscope	[33]
2017	MiSeq	TIRF microscope	[42]
2017–2019	MiSeq	Repurposed GA IIx	[32,52,53,54,55,56]
2019	NextSeq 500	Epifluorescence microscope	[29]
2020	MiSeq	MiSeq	[28]

HiTS: high-throughput sequencing; FLIP: fluorescent ligand interaction profiling; GA IIx: Genome Analyzer IIx; TIRF: total internal reflection fluorescence.

## Supplementary Materials

The following are available online at https://www.mdpi.com/article/10.3390/ijms22179202/s1. Table S1: Comparison of the light sources of the Genome Analyzer IIx and current Illumina sequencing platforms. Table S2: Fluorophores used for HiTS-FLIP with their characteristics. Table S3. Aptamers selected with HiTS-FLIP. Figure S1: Illustration of Prot-MaP according to Layton et al. (a) The initial DNA library designed for sequencing, followed by in situ transcription as in RNA-MaP (see Figure 4) and translation. (b) The Prot-MAP process.
