# Supplementary material for: High-Throughput Selection and Characterisation of Aptamers on Optical Next-Generation Sequencers
Source: Int J Mol Sci. 2021 Aug 25;22(17):9202. doi: 10.3390/ijms22179202 (PMC8431662; doi:10.3390/ijms22179202)
Supplement: Supplementary file 1 [file ijms-22-09202-s001.zip › ijms-1320087-supplementary.pdf]

International Journal of
Molecular Sciences

MDPI

Review – Supplementary Materials

High-Throughput Selection and Characterisation of Aptamers on Next Generation Sequencers

Alissa Drees [1], Markus Fischer [1,2,*]

[1] Hamburg School of Food Science, Institute of Food Chemistry, University of Hamburg, Grindelallee 117, 20146 Hamburg, Germany
[2] Center for Hybrid Nanostructures (CHyN), Department of Physics, University of Hamburg, Luruper Chaussee 149, 22761 Hamburg, Germany
* Correspondence: markus.fischer@uni-hamburg.de

Table S1. Comparison of the light sources of the Genome Analyzer IIx and current Illumina sequencing platforms.

Sequencing Platform	Light Source	Fluorescence Excitation Wavelength	Reference
Genome Analyzer IIx	Laser	532 nm, 635 nm, and 660 nm	[1]
iSeq 100	LED	520 nm	[2]
MiniSeq	LED	515 nm, 650 nm	[3]
MiSeq	LED	530 nm, 660 nm	[4]
NextSeq 550	LED, Laser	550 nm, 650 nm 780 nm	[5]
NextSeq 1000/2000	Laser	449 nm, 523 nm, 820 nm	[6]
NovaSeq 6000	Laser	532 nm, 660 nm, 780 nm, 790 nm	[7]

LED: Light-emitting diodes.

Table S2. Fluorophores used for HiTS-FLIP with their characteristics.

Fluorophore	Excitation Maximum [nm]	Emission Maximum [nm]	Extinction Coefficient at Emission Maximum [$cm^{-1} M^{-1}$]	Quantum Yield	Lifetime [ns]	References for HiTS-FLIP	Reference
Alexa 488	495	519	73,000	0.92	4.1	[8]	[9]
Alexa 555	555	565	155,000	0.10	0.3	[10]	[9]
Alexa 647	650	668	270,000	0.33	1.0	[8,11–13]	[9]
Atto647	645	669	120,000	0.20	2.3	[8]	[14]
DyLight 550	562	576	150,000	-	-	[10]	[15]
DyLight 650	652	672	250,000	-	-	[13]	[15]
EGFP	488	507	55,900	0.60	2.6	[16,17]	[18]
mOrange	548	562	71,000	0.69	-	[16,17,19,20]	[21]
SNAP-Surface 549 fluor	560	595	-	-	-	[10,22,23]	[24]
Spinach + DFHBI	482	505	31,000	-	-	[25]	[26]
Cy3	554	568	150,000	0.15	2.8	[8,11–13,25,27]	[28]
Cy5	650	670	250,000	0.27	-	[25]	[28]

The given values may vary depending on the applied conditions.

Table S3. Aptamers selected with HiTS-FLIP.

Type of Aptamer	Type of Target	Pre-selection Rounds	Affinity	Reference
DNA	Cell	0	40-fold over background<br>$P = 1.2 \times 10^{-4}$	[29]
Base-modified DNA	Protein	6	K_d = 3 µM	[13]
Base-modified DNA	Peptide	1	K_d = 1.9 µM	[13]
Base-modified DNA	Protein	2	K_d = 2.8 nM	[13]
RNA	Protein	M	K_d = 1.0 nM (2.6 nM)	[22]
RNA	Protein	M	K_d = 0.17 nM (0.17 nM)	[10]
RNA	Protein	M	K_d = 0.01 nM (4.3 nM)	[17]
RNA	Protein	M	K_d = 0.47 nM (5.2 nM)	[17]
Peptide	Protein	M	LoD = 0.14 nM (1.1 nM)	[10]

M: Mutational assay; the affinity of the consensus sequence is given in brackets; LoD: Limit of Detection.

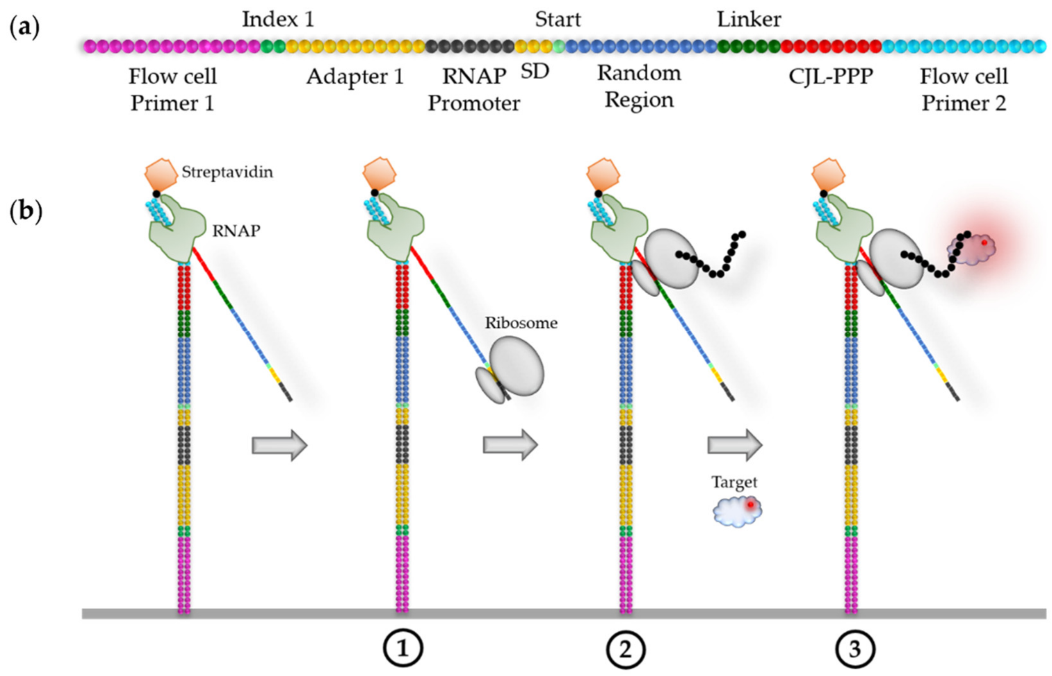

Figure S1. Illustration of Prot-MaP according to Layton et al. [10]. (**a**) The initial DNA library designed for sequencing, followed by in situ transcription as in RNA-MaP (see Figure 4) and translation. RNAP: RNA polymerase; SD: Shine-Dalgarno sequence; Start: Start codon; CJL-PPP: Ribosome stalling sequence encoding a modified polyproline motif. (**b**) The Prot-MAP process starting from the RNA display generated according to RNA-MaP (Figure 4b).

References

1. Illumina Inc. Genome Analyzer IIx System Specification Sheet. **2009**, 770-2009-017.
2. Illumina Inc. iSeq 100 Sequencing System Specification Sheet. **2018**, 770-2017-020-B
3. Illumina Inc. MiniSeq Sequencing System Specification Sheet. **2019**, 770-2015-039-C.
4. Illumina Inc. MiSeq-System Specification Sheet. **2021**, M-GL-00006 v1.0.
5. Illumina Inc. NextSeq 550 Sequencing System Specification Sheet. **2019**, 770-2013-053-F.
6. Illumina Inc. NextSeq 1000 and NextSeq 2000 Sequencing Systems Specification Sheet. **2021**, M-NA-00008 v1.0.
7. Illumina Inc. NovaSeq 6000 Sequencing System Specification Sheet. **2020**, 770-2016-025-N.

8. Jung, C.; Hawkins, J.A.; Jones Jr, S.K.; Xiao, Y.; Rybarski, J.R.; Dillard, K.E.; Hussmann, J.; Saifuddin, F.A.; Savran, C.A.; Ellington, A.D. Massively parallel biophysical analysis of CRISPR-Cas complexes on next generation sequencing chips. *Cell* **2017**, *170*, 35–47. e13.
9. Thermo Fisher Scientific Inc. Technical Notes and Product Highlights: The Alexa Fluor Dye Series—Note 1.1. https://www.thermofisher.com/de/de/home/references/molecular-probes-the-handbook/technical-notes-and-product-highlights/the-alexa-fluor-dye-series.html (accessed on 05.07.2021).
10. Layton, C.J.; McMahon, P.L.; Greenleaf, W.J. Large-scale, quantitative protein assays on a high-throughput DNA sequencing chip. *Molecular cell* **2019**, *73*, 1075–1082. e1074.
11. Boyle, E.A.; Andreasson, J.O.; Chircus, L.M.; Sternberg, S.H.; Wu, M.J.; Guegler, C.K.; Doudna, J.A.; Greenleaf, W.J. High-throughput biochemical profiling reveals sequence determinants of dCas9 off-target binding and unbinding. *Proceedings of the National Academy of Sciences* **2017**, *114*, 5461–5466.
12. Munzar, J.D.; Ng, A.; Juncker, D. Comprehensive profiling of the ligand binding landscapes of duplexed aptamer families reveals widespread induced fit. *Nature communications* **2018**, *9*, 1–15.
13. Wu, D.; Feagin, T.; Mage, P.; Rangel, A.; Wan, L.; Li, A.; Coller, J.; Eisenstein, M.; Pitteri, S.; Soh, H.T. Automated platform for high-throughput screening of base-modified aptamers for affinity and specificity. *bioRxiv* **2020**, 2020.04.25.060004.
14. ATTO-TEC GmbH. Product Information: ATTO 647. **2021**.
15. Thermo Fisher Scientific Inc. DyLight Fluors Technology and Product Guide. **2021**.
16. Ozer, A.; Tome, J.M.; Friedman, R.C.; Gheba, D.; Schroth, G.P.; Lis, J.T. Quantitative assessment of RNA-protein interactions with high-throughput sequencing–RNA affinity profiling. *Nature protocols* **2015**, *10*, 1212–1233.
17. Tome, J.M.; Ozer, A.; Pagano, J.M.; Gheba, D.; Schroth, G.P.; Lis, J.T. Comprehensive analysis of RNA-protein interactions by high-throughput sequencing–RNA affinity profiling. *Nature methods* **2014**, *11*, 683–688.
18. Cormack, B.P.; Valdivia, R.H.; Falkow, S. FACS-optimized mutants of the green fluorescent protein (GFP). *Gene* **1996**, *173*, 33–38.
19. Nutiu, R.; Friedman, R.C.; Luo, S.; Khrebtukova, I.; Silva, D.; Li, R.; Zhang, L.; Schroth, G.P.; Burge, C.B. Direct measurement of DNA affinity landscapes on a high-throughput sequencing instrument. *Nature biotechnology* **2011**, *29*, 659–664.
20. Wolowski, V.R. High-quality, high-throughput measurement of protein-DNA binding using HiTS-FLIP. lmu, 2016.
21. Shaner, N.C.; Campbell, R.E.; Steinbach, P.A.; Giepmans, B.N.; Palmer, A.E.; Tsien, R.Y. Improved monomeric red, orange and yellow fluorescent proteins derived from *Discosoma* sp. red fluorescent protein. *Nature biotechnology* **2004**, *22*, 1567–1572.
22. Buenrostro, J.D.; Araya, C.L.; Chircus, L.M.; Layton, C.J.; Chang, H.Y.; Snyder, M.P.; Greenleaf, W.J. Quantitative analysis of RNA-protein interactions on a massively parallel array reveals biophysical and evolutionary landscapes. *Nature biotechnology* **2014**, *32*, 562–568.
23. She, R.; Chakravarty, A.K.; Layton, C.J.; Chircus, L.M.; Andreasson, J.O.; Damaraju, N.; McMahon, P.L.; Buenrostro, J.D.; Jarosz, D.F.; Greenleaf, W.J. Comprehensive and quantitative mapping of RNA–protein interactions across a transcribed eukaryotic genome. *Proceedings of the National Academy of Sciences* **2017**, *114*, 3619–3624.
24. New England Biolabs GmbH, I. SNAP-Surface 549 Product information. **2021**.
25. Svensen, N.; Peersen, O.B.; Jaffrey, S.R. Peptide synthesis on a next-generation DNA sequencing platform. *Chembiochem: a European journal of chemical biology* **2016**, *17*, 1628.
26. Song, W.; Strack, R.L.; Svensen, N.; Jaffrey, S.R. Plug-and-play fluorophores extend the spectral properties of Spinach. *Journal of the American Chemical Society* **2014**, *136*, 1198–1201.
27. Jarmoskaite, I.; Denny, S.K.; Vaidyanathan, P.P.; Becker, W.R.; Andreasson, J.O.; Layton, C.J.; Kappel, K.; Shivashankar, V.; Sreenivasan, R.; Das, R. A quantitative and predictive model for RNA binding by human Pumilio proteins. *Molecular cell* **2019**, *74*, 966–981. e918.
28. Mujumdar, R.B.; Ernst, L.A.; Mujumdar, S.R.; Lewis, C.J.; Waggoner, A.S. Cyanine dye labeling reagents: sulfoindocyanine succinimidyl esters. *Bioconjugate chemistry* **1993**, *4*, 105–111.
29. Mamet, N.; Rusinek, I.; Harari, G.; Shapira, Z.; Amir, Y.; Lavi, E.; Zamir, A.; Borovsky, N.; Joseph, N.; Motin, M. Ab-initio discovery of tumoricidal oligonucleotides in a DNA sequencing machine. *bioRxiv* **2019**, 630830.
